# Crystal structure, Hirshfeld surface and computational study of 1-(9,10-dioxo-9,10-di­hydroanthracen-1-yl)-3-propano­ylthio­urea

**DOI:** 10.1107/S2056989022003127

**Published:** 2022-03-29

**Authors:** Kenechukwu J. Ifeanyieze, Bikimi B. Ayiya, Obinna C. Okpareke, Tatiana V. Groutso, Jonnie N. Asegbeloyin

**Affiliations:** a Department of Pure and Industrial Chemistry, University of Nigeria, Nsukka, Enugu State, Nigeria; b Universal College of Learning, Private Bag 11022, Palmerston North, New Zealand; c School of Chemical Sciences, the University of Auckland, New Zealand

**Keywords:** anthra­quinone, thio­urea, crystal structure, Hirshfeld surface, computational study

## Abstract

The title compound, C_18_H_14_N_2_O_3_S, crystallizes in the ortho­rhom­bic crystal system and *Pbca* space group. The thio­urea chromophore is planar to an r.m.s deviation of 0.032 Å with the thiol­ate sulfur atom being the most deviated. Bifurcated N—H⋯O intra­molecular hydrogen bonds result in an *S*(6) supra­molecular synthon. In the crystal, mol­ecules are linked by N—H⋯O inter­molecular hydrogen-bonding inter­actions and stabilized by C—H⋯π and π–π inter­actions. Hirshfeld surface analysis and fingerprint plot indicate the H⋯H inter­molecular contacts as the highest contributor to the overall surface contacts (38%) and this is supported by the high dispersive and electrostatic inter­action energies.

## Abstract 2

In the title compound, the thio­urea chromophore is planar to an r.m.s deviation of 0.032 Å with the thiol­ate sulfur atom being the most deviated. Bifurcated N—H⋯O intra­molecular hydrogen bonds result in an *S*(6) supra­molecular synthon. In the crystal, mol­ecules are linked by N—H⋯O inter­molecular hydrogen-bonding inter­actions and stabilized by C—H⋯π and π–π inter­actions.

## Chemical context

Anthra­quinones, a group of tricyclic aromatic organic compounds, are the largest group of natural and synthetic quinones. A large number of them are well-known natural pigments found in plants, lichens, and fungi (Duval *et al.*, 2016 ▸). These compounds exhibit important biological activities, including anti­tumor (Huang *et al.*, 2007 ▸; Murdock *et al.*, 1979 ▸, Shrestha *et al.*, 2014 ▸, 2015 ▸; Chien *et al.*, 2015 ▸), anti-inflammatory (Chien *et al.*, 2015 ▸; Khan *et al.*, 2011 ▸), diuretic (Chien *et al.*, 2015 ▸), anti­arthritic (Davis *et al.*, 1986 ▸), anti­fungal (Wuthi-udomlert *et al.*, 2010 ▸), anti­bacterial (Fosso *et al.*, 2012 ▸), anti­malarial (Winter *et al.*, 1996 ▸), anti­oxidant (Dave & Ledwani, 2012 ▸), anti­leukemic (Chang & Lee, 1984 ▸; Ismail *et al.*, 1997 ▸), anti­viral and anti-HIV properties (Alves *et al.*, 2004 ▸; Barnard *et al.*, 1992 ▸; Schinazi *et al.*, 1990 ▸; Schrader *et al.*, 2000 ▸). Some amino­anthra­quinone derivatives have also been reported to be good DNA inter­calators (Hande, 2008 ▸; Schrader *et al.*, 2000 ▸). The versatility of acyl thio­ureas stems from their ease of preparation and ability to introduce different functionalities, resulting in compounds with very inter­esting biological properties including anti­fungal (del Campo *et al.*, 2002 ▸, 2004 ▸), anti­tumor (Sacht & Datt, 2000 ▸; Sacht *et al.*, 2000 ▸; Hernández *et al.*, 2005 ▸), anti­viral, anti­bacterial, herbicidal, insecticidal and pharmacological activities (Binzet *et al.*, 2006 ▸; Saeed *et al.*, 2010 ▸). Recently, our research group reported the synthesis and crystal structures of a number of thio­urea derivatives (Asegbeloyin *et al.*, 2018 ▸, 2019 ▸; Okpareke *et al.*, 2020 ▸; 2022 ▸; Oyeka *et al.*, 2021 ▸). In a continuation of our series on thio­urea derivatives, we present herein the crystal structure, Hirshfeld surface and computational study of a new potential biologically active thio­urea derivative with an amino­anthra­quinone moiety. 
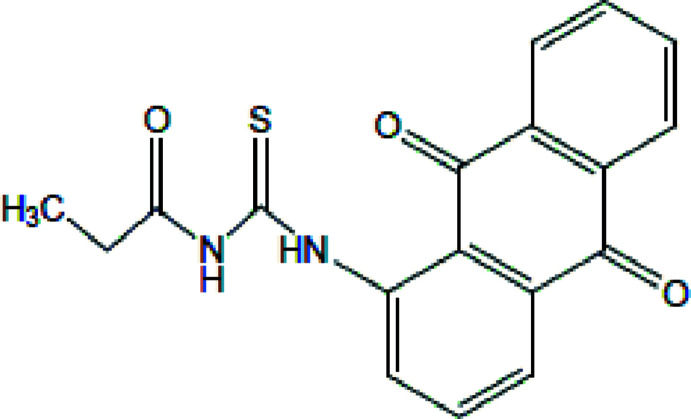


## Structural commentary

**Figure 1:**
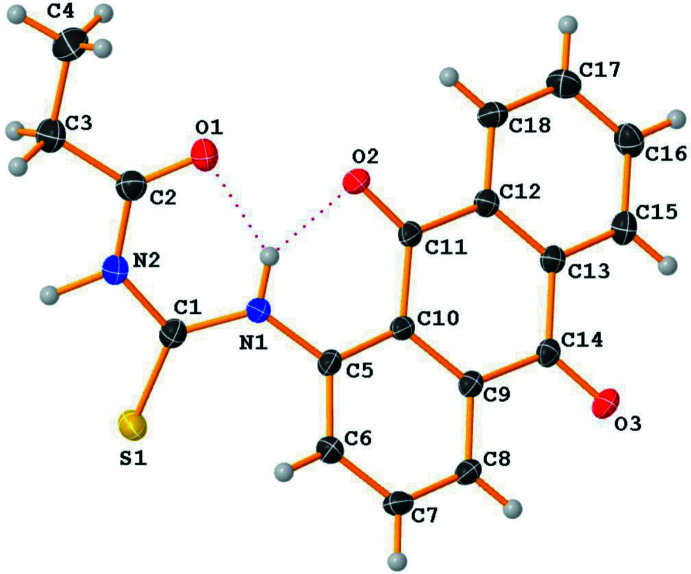
View of the mol­ecular structure of the title compound, with the atom labeling. Displacement ellipsoids are drawn at the 30% probability level. Intra­molecular hydrogen bonds are shown as dashed lines.

The title compound crystallizes in the ortho­rhom­bic crystal system and *Pbca* space group. The mol­ecular structure (Fig. 1 ▸) shows a central thio­urea chromophore flanked on either side by methyl­ene and anthra­quinone units. The central thio­urea moiety is essentially planar with an r.m.s deviation of 0.032 Å with the thiol­ate S atom being the most deviated out of the plane with a deviation of 0.044 (3) Å. The torsion angles between the thio­urea and the adjourning methyl­ene and anthra­quinone moieties are −177.5 (2) and −140.8 (2)°, respectively, indicating that the anthra­quinone moiety is slightly deviated from the thio­urea plane, compared to the methyl­ene moiety. The C1—N1—C5 bond angle of 126.09 (19)° subtended at the N1 atom is smaller than the less encumbered C2—N2—C1 angle [129.79 (19)°] subtended at N2 and larger than the central N1—C1—N2 [114.5 (2)°] bond angle subtended at the thiol­ate C1 carbon atom. The C1—N2 bond [1.395 (3) Å] is slightly longer than C1—N1 [1.364 (3) Å]. The thio­urea carbonyl oxygen and imine groups are involved in a strong intra­molecular N1—H1⋯O1 hydrogen bond (Table 1 ▸). The second amine nitro­gen N2 is also involved in a hydrogen-bonding *S*(6) graph-set (Kansiz *et al.*, 2022 ▸) inter­action.

## Supra­molecular features

**Figure 2:**
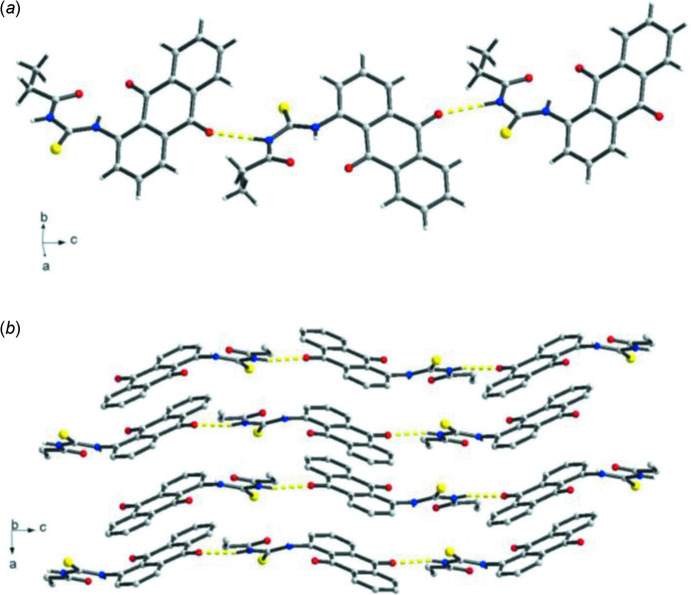
(*a*) Supra­molecular 1-D hydrogen-bonding inter­actions along *c-*axis direction of the title compound and (*b*) mol­ecular aggregation structure of the crystal along the *ab* plane, showing repeating units of pairwise π–π stacking inter­actions.

In the crystal, the mol­ecules are linked by imine N—H⋯O (anthra­quinone) hydrogen-bonding inter­actions, leading to supra­molecular chains running along the *c*-axis direction (Fig. 2 ▸ *a*). Supra­molecular layers are obtained from self-assembly of these chains *via* anthra­quinone π–π stacking inter­actions along the *ab* plane with centroid–centroid distances of 3.916(3), 3.531(5), 3.701(2) and 3.705(2) Å (Fig. 2 ▸ *b*). These inter­molecular inter­actions are balanced and stabilized by the phenyl C—H⋯O(carbonyl) and imine N—H⋯O(carbonyl) intra­molecular *S*(6) synthon.

## Hirshfeld surface analysis and fingerprint plots

**Figure 3:**
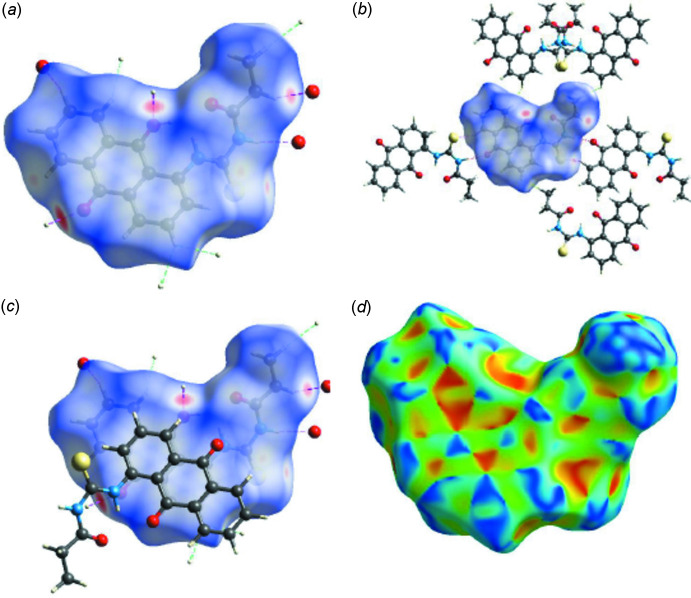
Hirshfeld surfaces mapped over (*a*), (*b*) and (*c*) *d*_norm_ and (*d*) shape-index showing inter­molecular atom-to-atom and π–π inter­actions in the crystal structure.

Hirshfeld surfaces (HS) and corresponding two-dimensional fingerprint plots (FPs) were calculated using the *Crystal Explorer 17.5* software (Turner *et al.*, 2017 ▸). The Hirshfeld surfaces mapped over *d*_norm_ and shape-index were generated according to a procedure described by Tan *et al.* (2019 ▸) and used for further analysis of the inter­molecular inter­actions. The HS mapped over *d*_norm_ shows the most intense red regions around the thio­urea N—H groups resulting from the amine-N—H⋯O (anthra­quinone) hydrogen-bonding inter­actions (Fig. 3 ▸ *a*). Other intense red spots can be identified around the thio­urea carbonyl oxygen and resulting from carbonyl C17—H17⋯O12 inter­molecular inter­action. Apart from the intense red spots, there are a number of other less intense red spots found around the alkyl C3 atom resulting from C3—H3*B*⋯O2 inter­molecular inter­action. Other inter­molecular inter­actions in the Hirshfeld surface are the anthra­quinone C—H⋯S(thio­urea) and anthra­quinone-C—H⋯H(alk­yl) inter­actions shown respectively as pink and green dotted lines in Fig. 3 ▸ *b*. The anthra­quinone π–π inter­actions can be seen in Fig. 3 ▸ *c*. The C⋯H/H⋯C contacts in the mol­ecule are responsible for the mol­ecular packing in the supra­molecular structure and are the result of the **C—H⋯π** and π–π inter­actions (Tan & Tiekink, 2020 ▸) and are depicted by mapping the structure over the shape-index isosurface as shown in Fig. 3 ▸ *d*. The C—H⋯π inter­actions appear as hollow orange areas (π⋯H—C) and bulging blue areas (C—H⋯π) in the compound. The small blue regions surrounding a bright orange spot within the anthro­quinone rings of the mol­ecule indicate π–π stacking inter­actions.

**Figure 4:**
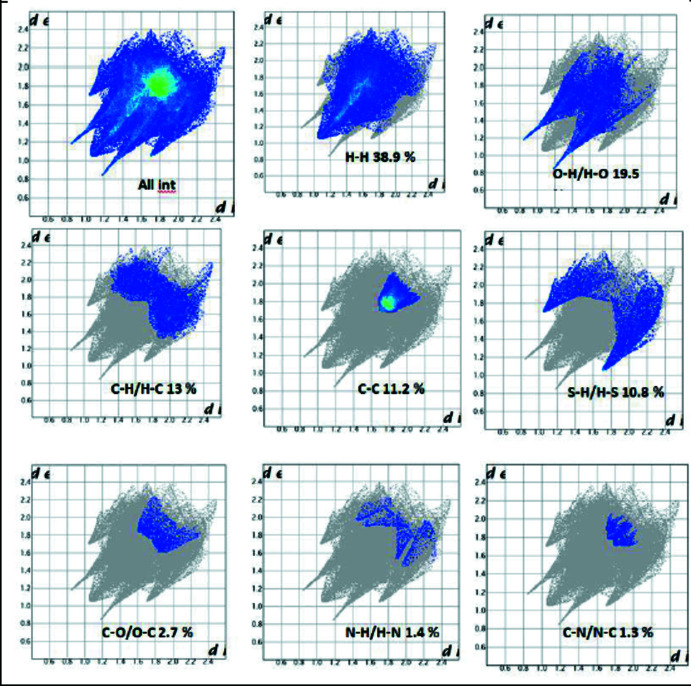
The overall and individual two-dimensional fingerprint plots for inter­molecular contacts in the crystal structure.

The overall two-dimensional fingerprint plot (Spackman & McKinnon, 2002 ▸; Tan & Tiekink, 2020 ▸) and those delineated into H⋯H, H⋯O/O⋯H, H⋯C/C⋯H, C⋯C, S⋯H/H⋯S and C⋯O/O⋯C inter­actions are illustrated in Fig. 4 ▸, and their percentage contributions are presented in Table 2 ▸. The overall fingerprint plot comprises all inter­molecular contacts in the mol­ecule and exhibits a shield-like profile with two symmetric spikes on each side of a triangular protrusion. These spikes are also observed in the fingerprint plots for the O⋯H/H⋯O contacts, which make a 19.5% contribution to the overall surface contact, but not in the other surface contacts. These spikes are due to the C—H⋯O and N2—H2⋯O3 hydrogen-bonding inter­actions in the crystal structure of the title compound. H⋯H contacts are the single highest contributor to the overall surface with a 38.0% contribution and and result from C—H⋯H and H⋯H dispersion inter­actions. The other major surface contacts are C⋯H/H⋯C (13.0%) S⋯H/H⋯S (10.8%), and C⋯C (11.2%), showing that C⋯H and π inter­molecular contacts contribute significantly to the overall stability of the supra­molecular architecture in the crystal structure (Ekowo *et al.*, 2020 ▸; Izuogu *et al.*, 2020 ▸).

## Inter­action energy calculations

**Figure 5:**
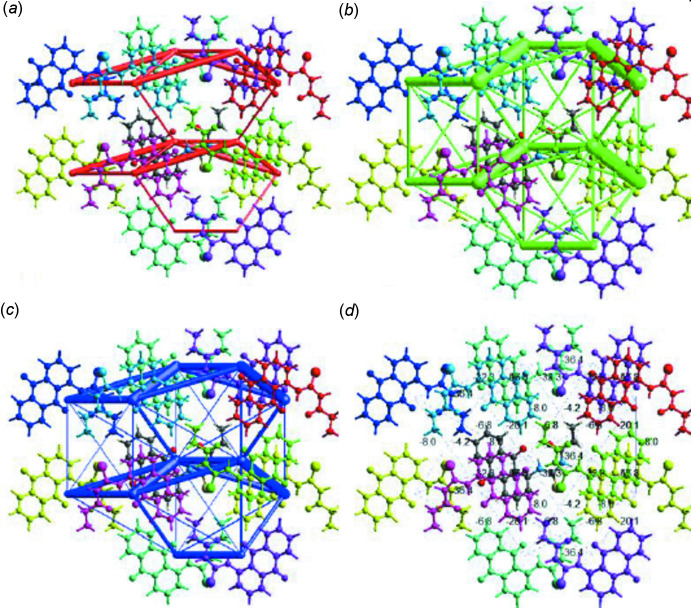
Perspective views of the energy frameworks of the title compound showing (*a*) electrostatic, (*b*) dispersion, (*c*) total energy and (*d*) annotated total energy. The cylindrical radius is proportional to the relative strength of the corresponding energies and they were adjusted to the same scale factor of 100 with a cut-off value of 5 kJmol^−1^ within 2 x 2 x 2 unit cells.

The inter­action energies between pairs of mol­ecules within the crystal of the title compound were calculated by adding up the four energy components, *viz*. electrostatic (*E*_ele_), polarization (*E*_pol_), dispersion (*E*_dis_), and exchange repulsion (*E*_rep_) (Tan *et al.*, 2019 ▸; Ayiya & Okpareke, 2021 ▸). The energies were obtained by calculating the wave function of each pair of mol­ecules or atoms at the B3LYP/6-31G(d,p) level of theory (Ayiya & Okpareke, 2021 ▸; Izuogu *et al.*, 2020 ▸). Qu­anti­tative estimations of the strength and nature of the inter­molecular inter­actions in title compound crystal with individual energy components (*E*_ele_, *E*_pol_, *E*_dis_, and *E*_rep_) as well as the sum of the energy components *E*_tot_ are presented in Table 3 ▸. This shows that the dispersive component of the energy makes the most significant contribution to the total inter­action energy profile in the crystal structure, probably due to the inter­molecular dispersive π inter­actions resulting from the π–π stacking of adjacent anthra­quinone ring systems in the crystal. The electrostatic component is the second highest contributor to the total inter­action energy and probably results from the C⋯H, H⋯H and van der Waals inter­actions. A graphical representation of the magnitude of the inter­action energies is presented in Fig, 5*a*–*d* in the form of energy frameworks to show the supra­molecular architecture using cylindrical poles joining the centroids of mol­ecular pairs. The red, green, and blue color-coded frameworks in Fig. 5 ▸ *a*, 5*b*, and 5*c*, respectively, represent the *E*_ele_, *E*_dis_, and *E*_tot_, energy components for inter­molecular inter­actions in crystal of the title compound, while Fig. 5 ▸ *d* shows the annotated *E*_tot_ energy. The magnitude of the cylindrical pipes indicates the significance of the *E*_ele_ energy component to the total inter­action energy and the mol­ecular packing in the crystal.

## Database survey

Anthra­quinones derivatives with thio­urea unit are scarce and our search for the basic architecture of the compound in the Cambridge Structural Database (CSD, version 5.42, update of May 2021; Groom *et al.*, 2016 ▸) did not reveal any structure similar to the title compound.

## Synthesis and crystallization

A solution of propionyl chloride (1.85 g, 0.02 mol) dissolved in 40 mL acetone was mixed with 30 mL of an acetone solution of potassium thio­cyanate (1.94 g, 0.02 mol). The reaction mixture was refluxed for 30 min to give a suspension of propionyliso­thio­cyanate, which was left to cool to room temperature. 1-Amino­anthra­quinone (4.47 g, 0.02 mol) was dissolved in 40 mL of acetone and the resulting solution was mixed with the suspension of propionyliso­thio­cyanate, and the mixture was stirred for 2 h. The resultant reddish suspension was filtered, and left at room temperature for 96 h to obtain a reddish crystalline solid of the title compound.

## Refinement

Crystal data, collection and structure refinement details are summarized in Table 4 ▸. The carbon-bound H atoms were placed in calculated positions and were included in the refinement using the riding-model approximation with *U*_iso_(H) set to 1.2*U*_eq_(C). The nitro­gen-bound H atoms were located in the difference-Fourier maps and refined freely with appropriate RIGU restraints placed on the bonds.

## Supplementary Material

Crystal structure: contains datablock(s) I. DOI: 10.1107/S2056989022003127/zn2016sup1.cif

Supporting information file. DOI: 10.1107/S2056989022003127/zn2016Isup2.cml

CCDC reference: 2161135

Additional supporting information:  crystallographic information; 3D view; checkCIF report

## Figures and Tables

**Table 1:** Hydrogen-bond geometry (Å, °)

*D*—H⋯*A*	*D*—H	H⋯*A*	*D*⋯*A*	*D*—H⋯*A*
N1—H1⋯O1	0.86	1.98	2.685 (2)	138
N1—H1⋯O2	0.86	2.14	2.652 (2)	117
N2—H2⋯O3^i^	0.86	2.19	3.038 (2)	167
C3—H3*B*⋯O2^ii^	0.97	2.52	3.414 (3)	153
C15—H15⋯S1^iii^	0.93	2.87	3.553 (2)	131
C17—H17⋯O2^iv^	0.93	2.47	3.280 (3)	145

Symmetry codes: (i) 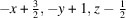 ; (ii) 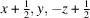 ; (iii) 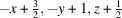 ; (iv) 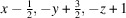 .

**Table 2:** Percentage contributions of inter­molecular contacts to the Hirshfeld surface

Contact	Percentage contribution
H⋯H	38.0
H⋯O/O⋯H	19.5
C⋯H/H⋯C	13.0
C⋯C	26.3
H⋯H	11.2
S⋯H/H⋯S	10.8
C⋯O/O⋯C	2.7
N⋯H/H⋯N	1.4
C⋯O/O⋯C	1.3

**Table 3:** A summary of the calculated inter­action energies for the title compound (kJ mol^−1^) Please define *N* and *R*

*N*	Symop	*R*	*E*_ele	*E*_pol	*E*_dis	*E*_rep	*E*_tot
1	*x*, −*y* + 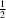 , *z* + 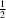	14.92	0.6	−0.2	−2.7	0.4	−1.6
0	-*x*, −*y*, −*z*	6.11	−24.1	−4.8	−85.9	77.8	−55.8
0	-*x* + 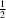 , −*y*, *z* + 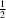	11.23	−33.2	−7.5	−17.8	38.4	−32.3
1	-*x* + 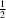 , −*y*, −*z* + 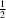	7.82	−17.7	−6.2	−44.9	42.1	−36.4
0	-*x* + 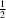 , *y* + 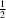 , *z*	9.48	−0.7	−1.1	−13.3	8.2	−8.0
0	*x* + 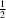 , −*y* + 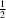 , −*z*	8.88	−10.8	−3.0	−17.6	14.2	−20.1
0	*x*, −*y* + 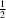 , *z* + 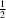	13.01	−0.0	−0.5	−9.9	3.6	−6.8
1	-*x*, *y* + 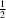 , −*z* + 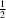	12.22	−0.1	−0.7	−10.2	8.5	−4.2
0	-*x*, −*y*, −*z*	5.85	−11.3	−1.1	−69.5	42.1	−47.3

**Table 4:** Experimental details

Crystal data	
Chemical formula	C_18_H_14_N_2_O_3_S
*M*_r_	338.37
Crystal system, space group	Orthorhombic, *P* *b* *c* *a*
Temperature (K)	100
*a*, *b*, *c* (Å)	7.3003 (1), 18.9557 (3), 21.9045 (3)
*V* (Å^3^)	3031.19 (8)
*Z*	8
Radiation type	Cu *K*α
μ (mm^−1^)	2.07
Crystal size (mm)	0.18 × 0.12 × 0.08

Data collection	
Diffractometer	XtaLAB Synergy, Dualflex, Pilatus 200K
Absorption correction	Multi-scan (*CrysAlis PRO*; Rigaku OD, 2018 ▸)
*T*_min_, *T*_max_	0.869, 1.000
No. of measured, independent and observed [*I* > 2σ(*I*)] reflections	18022, 3013, 2816
*R*_int_	0.034
(sin θ/λ)_max_ (Å^−1^)	0.624

Refinement	
*R*[*F* ^2^ > 2σ(*F* ^2^)], *wR*(*F* ^2^), *S*	0.048, 0.144, 1.13
No. of reflections	3013
No. of parameters	218
H-atom treatment	H-atom parameters constrained
Δρ_max_, Δρ_min_ (e Å^−3^)	0.67, −0.64

Computer programs: *CrysAlis PRO* (Rigaku OD, 2018 ▸), *SHELXT* (Sheldrick, 2015*a* ▸), *SHELXL* (Sheldrick, 2015*b* ▸), and *OLEX2* (Dolomanov *et al.*, 2009 ▸).

## supplementary crystallographic information

### Crystal data

**Table:**

C_18_H_14_N_2_O_3_S	*D*_x_ = 1.483 Mg m^−^^3^
*M**_r_* = 338.37	Cu *K*α radiation, λ = 1.54184 Å
Orthorhombic, *P**b**c**a*	Cell parameters from 10712 reflections
*a* = 7.3003 (1) Å	θ = 4.0–74.2°
*b* = 18.9557 (3) Å	µ = 2.07 mm^−^^1^
*c* = 21.9045 (3) Å	*T* = 100 K
*V* = 3031.19 (8) Å^3^	Block, clear colourless
*Z* = 8	0.18 × 0.12 × 0.08 mm
*F*(000) = 1408	

### Data collection

**Table:**

XtaLAB Synergy, Dualflex, Pilatus 200K diffractometer	3013 independent reflections
Radiation source: micro-focus sealed X-ray tube, PhotonJet (Cu) X-ray Source	2816 reflections with *I* > 2σ(*I*)
Mirror monochromator	*R*_int_ = 0.034
ω scans	θ_max_ = 74.3°, θ_min_ = 4.0°
Absorption correction: multi-scan (CrysAlisPro; Rigaku OD, 2018)	*h* = −8→8
*T*_min_ = 0.869, *T*_max_ = 1.000	*k* = −23→22
18022 measured reflections	*l* = −26→26

### Refinement

**Table:**

Refinement on *F*^2^	Primary atom site location: dual
Least-squares matrix: full	Hydrogen site location: inferred from neighbouring sites
*R*[*F*^2^ > 2σ(*F*^2^)] = 0.048	H-atom parameters constrained
*wR*(*F*^2^) = 0.144	*w* = 1/[σ^2^(*F*_o_^2^) + (0.0724*P*)^2^ + 3.6939*P*] where *P* = (*F*_o_^2^ + 2*F*_c_^2^)/3
*S* = 1.13	(Δ/σ)_max_ < 0.001
3013 reflections	Δρ_max_ = 0.67 e Å^−^^3^
218 parameters	Δρ_min_ = −0.64 e Å^−^^3^
0 restraints	

### Special details

**Table:**

Geometry. All esds (except the esd in the dihedral angle between two l.s. planes) are estimated using the full covariance matrix. The cell esds are taken into account individually in the estimation of esds in distances, angles and torsion angles; correlations between esds in cell parameters are only used when they are defined by crystal symmetry. An approximate (isotropic) treatment of cell esds is used for estimating esds involving l.s. planes.

### Fractional atomic coordinates and isotropic or equivalent isotropic displacement parameters (Å^2^)

**Table:**

	*x*	*y*	*z*	*U*_iso_*/*U*_eq_	
S1	0.72292 (8)	0.44246 (3)	0.25812 (3)	0.02439 (19)	
O2	0.7597 (2)	0.64440 (8)	0.41206 (7)	0.0228 (4)	
O3	0.6789 (2)	0.46534 (8)	0.60029 (7)	0.0244 (4)	
O1	0.9337 (3)	0.66694 (9)	0.29076 (7)	0.0287 (4)	
N1	0.8521 (3)	0.53993 (9)	0.33766 (8)	0.0195 (4)	
H1	0.889018	0.582673	0.342345	0.023*	
N2	0.8371 (3)	0.57086 (10)	0.23530 (8)	0.0223 (4)	
H2	0.815839	0.557602	0.198428	0.027*	
C12	0.6591 (3)	0.62664 (11)	0.51256 (9)	0.0171 (4)	
C5	0.8416 (3)	0.49780 (11)	0.39044 (9)	0.0172 (4)	
C13	0.6390 (3)	0.58121 (11)	0.56248 (9)	0.0169 (4)	
C10	0.7788 (3)	0.52594 (11)	0.44627 (9)	0.0154 (4)	
C11	0.7332 (3)	0.60171 (11)	0.45330 (9)	0.0163 (4)	
C9	0.7647 (3)	0.48093 (11)	0.49745 (9)	0.0159 (4)	
C6	0.9019 (3)	0.42781 (11)	0.38807 (10)	0.0200 (5)	
H6	0.951456	0.410016	0.352119	0.024*	
C7	0.8881 (3)	0.38493 (11)	0.43898 (10)	0.0208 (5)	
H7	0.927247	0.338318	0.436683	0.025*	
C15	0.5707 (3)	0.60672 (12)	0.61753 (10)	0.0220 (5)	
H15	0.556732	0.576471	0.650632	0.026*	
C8	0.8170 (3)	0.41032 (11)	0.49330 (10)	0.0179 (4)	
H8	0.804179	0.380524	0.526757	0.022*	
C14	0.6936 (3)	0.50620 (11)	0.55722 (9)	0.0172 (4)	
C18	0.6119 (3)	0.69770 (11)	0.51866 (10)	0.0223 (5)	
H18	0.625588	0.728232	0.485743	0.027*	
C2	0.8937 (3)	0.64023 (12)	0.24157 (10)	0.0230 (5)	
C17	0.5446 (3)	0.72278 (13)	0.57380 (11)	0.0262 (5)	
H17	0.513700	0.770149	0.577811	0.031*	
C1	0.8093 (3)	0.51926 (12)	0.27974 (10)	0.0203 (5)	
C16	0.5234 (3)	0.67731 (13)	0.62298 (11)	0.0254 (5)	
H16	0.477295	0.694251	0.659739	0.031*	
C3	0.8997 (4)	0.68035 (13)	0.18245 (11)	0.0298 (6)	
H3A	0.777627	0.681545	0.164961	0.036*	
H3B	0.978880	0.655647	0.154039	0.036*	
C4	0.9688 (5)	0.75570 (14)	0.19012 (13)	0.0380 (6)	
H4A	0.965684	0.779397	0.151405	0.057*	
H4B	1.092260	0.754860	0.205179	0.057*	
H4C	0.891940	0.780289	0.218617	0.057*	

### Atomic displacement parameters (Å^2^)

**Table:**

	*U*^11^	*U*^22^	*U*^33^	*U*^12^	*U*^13^	*U*^23^
S1	0.0304 (3)	0.0229 (3)	0.0198 (3)	−0.0057 (2)	0.0008 (2)	−0.0018 (2)
O2	0.0358 (9)	0.0158 (7)	0.0170 (7)	−0.0024 (6)	0.0019 (7)	0.0034 (6)
O3	0.0347 (10)	0.0221 (8)	0.0163 (8)	0.0002 (7)	0.0017 (6)	0.0062 (6)
O1	0.0417 (11)	0.0264 (9)	0.0180 (8)	−0.0020 (7)	−0.0021 (7)	−0.0015 (6)
N1	0.0294 (10)	0.0152 (8)	0.0140 (9)	−0.0018 (7)	0.0012 (7)	0.0009 (7)
N2	0.0289 (10)	0.0232 (10)	0.0148 (9)	−0.0010 (8)	−0.0003 (8)	−0.0001 (7)
C12	0.0153 (10)	0.0190 (10)	0.0168 (10)	−0.0003 (8)	−0.0012 (8)	0.0007 (8)
C5	0.0192 (11)	0.0175 (10)	0.0149 (10)	−0.0025 (8)	−0.0018 (8)	0.0025 (8)
C13	0.0142 (10)	0.0199 (10)	0.0165 (10)	−0.0004 (8)	−0.0019 (8)	0.0005 (8)
C10	0.0151 (10)	0.0146 (10)	0.0165 (10)	−0.0016 (7)	−0.0031 (8)	0.0013 (8)
C11	0.0168 (10)	0.0166 (10)	0.0156 (10)	−0.0022 (8)	−0.0032 (8)	0.0005 (8)
C9	0.0138 (10)	0.0178 (10)	0.0162 (10)	−0.0028 (8)	−0.0021 (8)	0.0014 (8)
C6	0.0224 (11)	0.0189 (10)	0.0187 (10)	0.0014 (8)	0.0004 (8)	−0.0010 (8)
C7	0.0240 (11)	0.0146 (10)	0.0239 (11)	0.0015 (9)	−0.0020 (9)	0.0013 (8)
C15	0.0208 (11)	0.0289 (12)	0.0164 (10)	0.0010 (9)	0.0004 (8)	0.0016 (9)
C8	0.0183 (10)	0.0164 (10)	0.0190 (10)	−0.0011 (8)	−0.0024 (8)	0.0042 (8)
C14	0.0158 (10)	0.0202 (10)	0.0156 (10)	−0.0026 (8)	−0.0033 (8)	0.0022 (8)
C18	0.0267 (11)	0.0174 (10)	0.0228 (11)	0.0025 (9)	0.0006 (9)	0.0015 (8)
C2	0.0235 (11)	0.0230 (11)	0.0226 (11)	0.0006 (9)	0.0019 (9)	0.0008 (9)
C17	0.0277 (12)	0.0211 (11)	0.0298 (12)	0.0043 (9)	0.0021 (10)	−0.0033 (9)
C1	0.0205 (11)	0.0218 (11)	0.0185 (10)	0.0013 (8)	0.0006 (8)	0.0017 (8)
C16	0.0258 (12)	0.0293 (12)	0.0212 (11)	0.0037 (9)	0.0028 (9)	−0.0048 (9)
C3	0.0398 (14)	0.0298 (13)	0.0199 (11)	−0.0015 (11)	0.0037 (10)	0.0016 (9)
C4	0.0559 (18)	0.0280 (13)	0.0302 (13)	−0.0085 (12)	0.0012 (12)	0.0085 (10)

### Geometric parameters (Å, º)

**Table:**

S1—C1	1.656 (2)	C9—C14	1.487 (3)
O2—C11	1.228 (3)	C6—H6	0.9300
O3—C14	1.226 (3)	C6—C7	1.384 (3)
O1—C2	1.226 (3)	C7—H7	0.9300
N1—H1	0.8600	C7—C8	1.384 (3)
N1—C5	1.407 (3)	C15—H15	0.9300
N1—C1	1.364 (3)	C15—C16	1.387 (3)
N2—H2	0.8600	C8—H8	0.9300
N2—C2	1.385 (3)	C18—H18	0.9300
N2—C1	1.395 (3)	C18—C17	1.388 (3)
C12—C13	1.400 (3)	C2—C3	1.503 (3)
C12—C11	1.484 (3)	C17—H17	0.9300
C12—C18	1.397 (3)	C17—C16	1.388 (3)
C5—C10	1.411 (3)	C16—H16	0.9300
C5—C6	1.399 (3)	C3—H3A	0.9700
C13—C15	1.392 (3)	C3—H3B	0.9700
C13—C14	1.481 (3)	C3—C4	1.524 (4)
C10—C11	1.482 (3)	C4—H4A	0.9600
C10—C9	1.412 (3)	C4—H4B	0.9600
C9—C8	1.395 (3)	C4—H4C	0.9600
			
C5—N1—H1	117.0	C9—C8—H8	120.3
C1—N1—H1	117.0	C7—C8—C9	119.49 (19)
C1—N1—C5	126.06 (19)	C7—C8—H8	120.3
C2—N2—H2	115.1	O3—C14—C13	121.5 (2)
C2—N2—C1	129.79 (19)	O3—C14—C9	120.32 (19)
C1—N2—H2	115.1	C13—C14—C9	118.13 (18)
C13—C12—C11	121.73 (19)	C12—C18—H18	120.0
C18—C12—C13	119.5 (2)	C17—C18—C12	120.1 (2)
C18—C12—C11	118.74 (19)	C17—C18—H18	120.0
N1—C5—C10	121.02 (18)	O1—C2—N2	123.4 (2)
C6—C5—N1	119.38 (19)	O1—C2—C3	122.8 (2)
C6—C5—C10	119.54 (19)	N2—C2—C3	113.80 (19)
C12—C13—C14	120.11 (19)	C18—C17—H17	119.9
C15—C13—C12	120.1 (2)	C18—C17—C16	120.1 (2)
C15—C13—C14	119.83 (19)	C16—C17—H17	119.9
C5—C10—C11	121.97 (18)	N1—C1—S1	127.30 (17)
C5—C10—C9	118.90 (19)	N1—C1—N2	114.5 (2)
C9—C10—C11	119.10 (19)	N2—C1—S1	118.19 (16)
O2—C11—C12	119.42 (19)	C15—C16—C17	120.3 (2)
O2—C11—C10	121.78 (19)	C15—C16—H16	119.9
C10—C11—C12	118.78 (18)	C17—C16—H16	119.9
C10—C9—C14	121.97 (18)	C2—C3—H3A	109.0
C8—C9—C10	120.52 (19)	C2—C3—H3B	109.0
C8—C9—C14	117.50 (18)	C2—C3—C4	112.9 (2)
C5—C6—H6	119.9	H3A—C3—H3B	107.8
C7—C6—C5	120.3 (2)	C4—C3—H3A	109.0
C7—C6—H6	119.9	C4—C3—H3B	109.0
C6—C7—H7	119.5	C3—C4—H4A	109.5
C6—C7—C8	121.0 (2)	C3—C4—H4B	109.5
C8—C7—H7	119.5	C3—C4—H4C	109.5
C13—C15—H15	120.0	H4A—C4—H4B	109.5
C16—C15—C13	119.9 (2)	H4A—C4—H4C	109.5
C16—C15—H15	120.0	H4B—C4—H4C	109.5

### Hydrogen-bond geometry (Å, º)

**Table:**

*D*—H···*A*	*D*—H	H···*A*	*D*···*A*	*D*—H···*A*
N1—H1···O1	0.86	1.98	2.685 (2)	138
N1—H1···O2	0.86	2.14	2.652 (2)	117
N2—H2···O3^i^	0.86	2.19	3.038 (2)	167
C3—H3*B*···O2^ii^	0.97	2.52	3.414 (3)	153
C15—H15···S1^iii^	0.93	2.87	3.553 (2)	131
C17—H17···O2^iv^	0.93	2.47	3.280 (3)	145

Symmetry codes: (i) −*x*+3/2, −*y*+1, *z*−1/2; (ii) *x*+1/2, *y*, −*z*+1/2; (iii) −*x*+3/2, −*y*+1, *z*+1/2; (iv) *x*−1/2, −*y*+3/2, −*z*+1.
